# Hedgehog Signal and Genetic Disorders

**DOI:** 10.3389/fgene.2019.01103

**Published:** 2019-11-08

**Authors:** Noriaki Sasai, Michinori Toriyama, Toru Kondo

**Affiliations:** ^1^Developmental Biomedical Science, Division of Biological Sciences, Nara Institute of Science and Technology, Ikoma, Japan; ^2^Systems Neurobiology and Medicine, Division of Biological Sciences, Nara Institute of Science and Technology, Ikoma, Japan; ^3^Department of Biomedical Chemistry, School of Science and Technology, Kwansei Gakuin University, Sanda, Japan; ^4^Division of Stem Cell Biology, Institute for Genetic Medicine, Hokkaido University, Sapporo, Japan

**Keywords:** sonic hedgehog (Shh), development, genetic disease, mouse model, ciliopathies, cancer

## Abstract

The hedgehog (Hh) family comprises sonic hedgehog (Shh), Indian hedgehog (Ihh), and desert hedgehog (Dhh), which are versatile signaling molecules involved in a wide spectrum of biological events including cell differentiation, proliferation, and survival; establishment of the vertebrate body plan; and aging. These molecules play critical roles from embryogenesis to adult stages; therefore, alterations such as abnormal expression or mutations of the genes involved and their downstream factors cause a variety of genetic disorders at different stages. The Hh family involves many signaling mediators and functions through complex mechanisms, and achieving a comprehensive understanding of the entire signaling system is challenging. This review discusses the signaling mediators of the Hh pathway and their functions at the cellular and organismal levels. We first focus on the roles of Hh signaling mediators in signal transduction at the cellular level and the networks formed by these factors. Then, we analyze the spatiotemporal pattern of expression of Hh pathway molecules in tissues and organs, and describe the phenotypes of mutant mice. Finally, we discuss the genetic disorders caused by malfunction of Hh signaling-related molecules in humans.

## Hedgehog Genes and Signal Transduction

In vertebrates, at least three hedgehog (Hh)-related genes have been reported (Ingham and McMahon, 2001). The three Hh genes identified in mice and in humans are *sonic hedgehog* (*Shh*), *desert hedgehog* (*Dhh*), and *Indian hedgehog* (*Ihh*), which are similarly processed and secreted from cells, and evoke common signaling pathways in receiving cells (Ingham and McMahon, 2001).

Once produced, Hh polypeptides are cleaved at the intermediate site, and the amino-terminal part is functional as the signal molecules. After processed by cholesterol and palmitate, the protein is secreted from the producing cells and binds to the membrane protein Patched (Ptch). The signal is received by another membrane protein, smoothened (Smo), and then transduced into the nucleus by the transcription factor Gli. Gli stability and activity are modified by the kinesin-like protein KIF7 and the scaffold protein suppressor of fused (SuFu).

**Figure 1:**
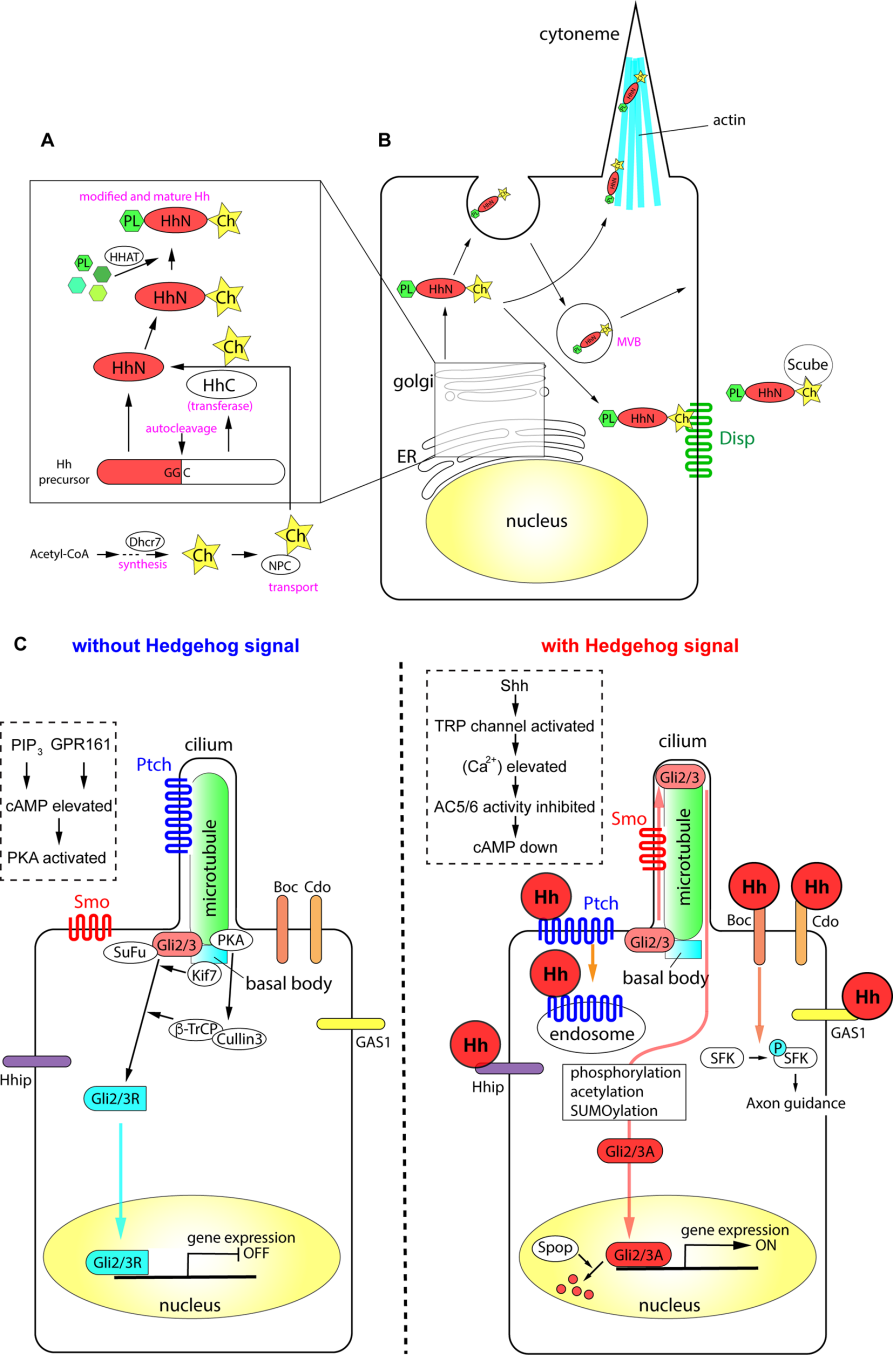
The processing of Hh proteins and their intracellular signaling pathways. **(A)** Cleavage of Hh polypeptides and their modifications in ER and golgi apparatus. **(B)** Processing, modifications and transport of Hh proteins in the producing cells. **(C)** Active signaling pathways in absence and in presence of Hh molecules. Schemas in dotted rectangles represent regulation of cAMP level in the cilia. The figure was made based on the information in (Varjosalo and Taipale, 2008; Balaskas et al., 2012; Moore et al., 2016; Parchure et al., 2018).

In the first part of this review, we discuss the production and secretion of Hh proteins in detail, and introduce the mediators involved in protein modifications and signaling pathways. The mechanisms are summarized in **Figure 1**.

### Production and Secretion of Hh Proteins

All Hh polypeptides produced are transported into the endoplasmic reticulum (ER) and Golgi apparatus, where they undergo autoprocessing (Jeong and McMahon, 2002; Cai and Liu, 2016). The polypeptides are cleaved into two parts, the amino-terminal domain, which functions as a signaling molecule, and the carboxyl-terminal domain, which functions in autoprocessing regulation (Mann and Beachy, 2004). The amino-terminal part is further modified with palmitate and cholesterol (Porter et al., 1996; Chamoun et al., 2001; Lewis et al., 2001; Mann and Beachy, 2004; Chen et al., 2004a; Chen et al., 2011a; Li et al., 2006; Hardy and Resh, 2012). The carboxyl ends of the initial polypeptides act as cholesterol transferases (Porter et al., 1996), and palmitoylation is mediated by skinny Hh (Ski) acyltransferase (also known as Hh acyltransferase or HHAT), a transmembrane acyltransferase located in the ER (Chamoun et al., 2001; Dennis et al., 2012; Konitsiotis et al., 2015). The modified Shh protein was recently purified, and the fatty acids bound to the amino terminus were identified, which revealed that other unsaturated fatty acids in addition to palmitate are involved in the modification (Long et al., 2015). These lipid modifications are essential for the stability of the protein in the extracellular matrix (Callejo et al., 2006) and during long-range transport (Lewis et al., 2001; Zeng et al., 2001). The Niemann-Pick C (NPC1/2) proteins transport cholesterol from the endosome to the ER (Loftus et al., 1997; Tukachinsky et al., 2012; Formichi et al., 2018), and play an essential role in cholesterol modification of Hh. The membrane protein dispatched (Disp) and the secreted protein Scube2 bind to the cholesterol moiety of the modified Hh proteins (Tukachinsky et al., 2012) and release them from the cell membrane.

Some of the Hh proteins at the cell surface can be recycled. After recycling, the Hh proteins are released by lipid bilayer membrane vesicles called exosomes (Vlassov et al., 2012; Parchure et al., 2018). Exosomes are formed at the multivesicular body (MVB) in the cytosol, and bud from the plasma membrane with a size of 30–100 nm containing functional molecules (Parchure et al., 2018). For exosome formation, membrane proteins collectively called endosomal sorting complexes required for transport (ESCRT) are essential (Juan and Furthauer, 2018).

A specialized filopodial structure, the cytoneme, which forms in Hh-producing cells, is important for transmission of the Hh signal. The cytoneme was initially identified in flies (Bischoff et al., 2013; Gonzalez-Mendez et al., 2017), and later reported in the chick embryonic limb bud (Panman and Zeller, 2003; Sanders et al., 2013). The presence of microvesicles and the cytoneme, together with the palmitoylation and cholesterol modification of Hh proteins, mediate the transport of the Hh signal to distant parts in the tissues (Chen et al., 2004a; Sanders et al., 2013; D'Angelo et al., 2015).

Overall, the efficient production and secretion of active Hh proteins involves the following: (i) processing of the polypeptides by autocleavage, (ii) modification of the polypeptides by cholesterol and palmitate, (iii) recycling and packaging of the proteins in the microvesicles, and (iv) the presence of cytosolic structures including cytonemes.

### Membrane Receptors in Hh-Responding Cells

When Hh proteins reach the surface of a responding cell, the signals are introduced into the cell by two membrane proteins, Ptch and Smo. Ptch, a receptor protein that directly interacts with Hh proteins (Marigo et al., 1996; Motoyama et al., 1998b), is a 12-span membrane protein that localizes to the ciliary membrane in the absence of Hh (Rohatgi et al., 2007). Ptch binds to Hh, forming a complex that moves from primary cilia to the plasma membrane. In the case of Shh, the Ptch-Shh complex undergoes endocytosis, which requires the membrane-remodeling GTPase dynamin (Incardona et al., 2000; Ferguson and De Camilli, 2012) and the HECT-domain ubiquitin E3 ligases Smurf1/2 (Yue et al., 2014), and leads to degradation in the lysosome. Once Ptch is cleared from the ciliary membrane, the G protein-coupled receptor (GPCR) Smo is translocated from the plasma membrane to the cilium (Corbit et al., 2005). During this process, Smo is phosphorylated at specific serine residues in the carboxyl intracellular region by casein kinase 1α (CK1α) and G protein-coupled receptor kinase 2 (GRK2) (Chen et al., 2004b; Chen et al., 2011b), and interacts with β-arrestin 2 (Chen et al., 2004b). Smo further forms a complex with the kinesin-like protein Kif3a, and is translocated into primary cilia (Kovacs et al., 2008). β-Arrestin 2 is essential for the translocation of Smo into cilia (Kovacs et al., 2008), and contributes to the dynamin-dependent internalization of Smo (Chen et al., 2004b). The role of β-arrestin 2-mediated internalization of the signaling molecule (Ma and Pei, 2007), together with its involvement in the translocation of Smo, suggests that β-arrestin 2 regulates the activity and the stability of the Smo protein.

The Ptch polypeptide contains a sterol sensing domain (Martin et al., 2001; Strutt et al., 2001) and binds to cholesterol. The interaction of Ptch and cholesterol is necessary for pumping out cholesterol molecules from the endosome or from the plasma membrane (Bidet et al., 2011). In the absence of Hh, Ptch pumps out the cholesterol molecule (Denef et al., 2000; Khaliullina et al., 2009; Blassberg and Jacob, 2017). By contrast, when cells are exposed to Hh, Ptch is transported into the endosome and degraded (Incardona et al., 2000; Yue et al., 2014), and Smo is modified with cholesterol. Modified Smo activates cytosolic phospholipase A2 to release a fatty acid, arachidonic acid, and the interaction between arachidonic acid and Smo promotes the ciliary localization of Smo through allosteric changes (Arensdorf et al., 2017). Altogether, the cholesterol modification of Smo is essential for its complete activity and for the localization of the protein (Blassberg et al., 2016; Huang et al., 2016; Luchetti et al., 2016).

### Intracellular Signaling Pathways and Gli Transcription Factors

Activation of the Hh signal at the membrane level leads to its transduction into the nucleus by the transcription factors Gli2/3. Gli2/3 are Krüppel-like transcription factors containing zinc-finger DNA-binding domains with dual activity: the amino terminus contains a transcriptional repressor domain and the carboxyl terminus has an activator domain. In the absence of the extracellular Hh ligand, the synthesized Gli2/3 polypeptides form complexes with protein kinase A (PKA), CK1, and glycogen synthase kinase 3 (GSK3), the latter two of which are serine-threonine protein kinases (Tempe et al., 2006). This complex interacts with the scaffold protein SuFu in the absence of the Hh signal. The role of SuFu is to stabilize the Gli2/3 proteins (Chen et al., 2009; Humke et al., 2010); in the absence of SuFu, both Gli2/3 proteins are drastically destabilized and become undetectable by western blotting (Chen et al., 2009; Humke et al., 2010). Activation of the Hh signaling pathway disrupts the interaction between SuFu and Gli (Tukachinsky et al., 2010).

In the absence of an extracellular Hh ligand, the calcium level in the cytosol and in the cilium is relatively high (Moore et al., 2016). Gli2/3 are phosphorylated by PKA and subsequently targeted by the Skp, cullin, F-box (SCF) ubiquitin ligase complex, which is composed of the scaffold protein cullin 3 and the E3-type ubiquitin ligase β-Trcp (Bhatia et al., 2006; Wang and Li, 2006). The Gli2/3 proteins are cleaved at the amino terminus, and the truncated proteins with the transcriptional repressor domains (Gli2/3R; R for "repressive") are translocated into the nucleus to bind to DNA target sites (Svard et al., 2006; Chen et al., 2009).

As Smo couples with Gi-type G proteins (Riobo et al., 2006), the exposure of cells to Hh (i.e., activation of Smo) reduces PKA activity (Moore et al., 2016), which decreases cAMP levels. Thus, the newly synthesized Gli2/3 polypeptides are not cleaved, and the full-length polypeptides are maintained. These Gli2/3 polypeptides are further modified by sumoylation, phosphorylation, and acetylation (Cox et al., 2010; Humke et al., 2010; Coni et al., 2013; Niewiadomski et al., 2014), and translocated into the nucleus to act as transcriptional activators (Gli2/3A; A for “active”).

In the nucleus, the representative DNA sequence for Gli2/3 is GACCACCCA/TGGGTGGTC (Sasaki et al., 1997). However, recent chromatin immunoprecipitation (ChIP) and computational prediction studies identified several other sequences with different affinities (Vokes et al., 2007; Vokes et al., 2008; Oosterveen et al., 2012; Peterson et al., 2012).

Compared with Gli2/3R, Gli2/3A are relatively labile, and are ubiquitinated at the nucleus by the E3 ubiquitin ligase adaptor speckle-type POZ protein (Spop) (Humke et al., 2010; Cai and Liu, 2017).

Target genes of the Hh signaling pathway are cell type-dependent. In most cells responsive to Hh, the common target genes are Ptch and Gli1 (Stamataki et al., 2005; Cohen et al., 2015; Tickle and Towers, 2017). In the developing neural tube, the Nkx6.1, Olig2, Nkx2.2, and FoxA2 genes (Vokes et al., 2007; Kutejova et al., 2016), which confer ventral neural fates, have Gli-binding sites in their regulatory regions (mentioned in detail later).

### Other Hh Receptors

Additional Hh receptors that act in concert with Ptch and/or Smo have been identified and characterized. Hh-interacting protein (Hhip) is a single-pass membrane glycoprotein with epidermal growth factor (EGF)-repeat domains that binds to the three Hh proteins with similar affinity to Ptch (Chuang and McMahon, 1999). Although the downstream pathway remains to be fully elucidated, Hhip overexpression blocks Shh signaling activity, suggesting that Hhip is a negative regulator of Hh signaling (Bishop et al., 2009; Kwong et al., 2014). However, this effect occurs in a non-cell autonomous manner. The Hhip protein is released from producing cells and directly binds to Shh to block signaling (Kwong et al., 2014). On the other hand, in Shh-producing cells, Hhip gene expression is induced by the Hh signal, and the Hhip protein is internalized and degraded upon Smo activation (Kwong et al., 2014), thereby enabling cell autonomous Hh signaling. This complex regulatory loop may explain the temporal changes in Hh signaling activity (Dessaud et al., 2007; Balaskas et al., 2012). Other Hh receptor proteins identified include the glycosylphosphatidylinositol-anchored cell-surface protein growth arrest-specific 1 (Gas1), the membrane protein cell-adhesion molecule-related, downregulated by oncogenes (Cdo), and brother of Cdo (Boc) (Tenzen et al., 2006; Zhang et al., 2006; Allen et al., 2007; Allen et al., 2011). Cdo and Boc have immunoglobulin and fibronectin type III (FNIII) repeats in the extracellular domain, and possess structural properties similar to those of the axon guidance receptors Robo and deleted in colorectal cancer (Tessier-Lavigne and Goodman, 1996; Okada et al., 2006). Cdo and Boc function in axon guidance (Okada et al., 2006). In the developing chick limb, Cdo and Boc localize to discrete microdomains along extensive filopodial structures in the signal-receiving cells, and these receptor proteins interact with the filopodia (cytonemes) of Shh-producing cells (Sanders et al., 2013).

Hhip, Gas1, Cdo, and Boc bind to Shh directly. However, in contrast to Ptch, which localizes to cilia in the absence of Hh, these membrane proteins localize to the plasma membrane including filopodial structures. The distinct localization of the proteins may account for the variation in temporal and/or spatial Hh signaling activity. In summary, receptors in cilia and the plasma membrane interact with Hh proteins and transduce the signals into the nucleus, where Gli transcription factors act as effectors of Hh signaling.

## Functions of Hedgehog Proteins and Their Related Genes in Tissues

Hh plays an important role in many biological events, and mutant mice deficient in Hh-related genes therefore show various and severe phenotypes. Mutations, deficiencies, and abnormal expression levels of Hh proteins and related genes cause malformations, hyperplasia, and growth retardation in tissues, especially in the central nervous system (CNS), craniofacial structures, limbs, and skeleton at developmental stages. Conversely, persistent or aberrantly high Hh activation leads to tumorigenesis in organs at postnatal stages. In the next sections, we discuss the tissue distribution of Hh proteins, especially Shh, and the related genes. In addition, we provide data on the critical roles of these molecules in development and tissue homeostasis, which are mostly derived from studies in model mice. The principal gene knockout or mutant phenotypes in mice are summarized in **Table 1**.

**Table 1:** Main phenotypes and symptoms caused by the mutations and deficiencies of the Hh-related genes in mice and in humans.

Phenotypes in the mouse						Syndromes and symptoms in human	
Category	Gene	Gene product	Lethality	Main reported phenotype	Reports	Syndromes; symptoms	Reports
**signal molecules**	**Shh** **(Sonic Hedgehog)**	secreted factor	die by e9.5	cyclopia, defective axial patterning	Bulgakov et al., 2004; Chiang et al., 1996	holoprosencephary	Roessler et al., 1996
	**Dhh** **(Desert Hedgehog)**	secreted factor	viable	infertile; fail to produce mature spermatozoa	Clark et al., 2000	gonadal dysgenesis	Canto et al., 2004; Canto et al., 2005; Werner et al., 2015
	**Ihh** **(Indian Hedgehog)**	secreted factor	half of the KO mice die during e10.5-e12.5, and the rest die soon after birth	incomplete forelimb formation. Perinatally die due to the problem of the respiratory system	St-Jacques et al., 1999	Cleft lip and palate, brachydactyly (shortning of fingers and toes) type A-1	Gao et al., 2001
**modification and** **secretory pathway**	**HHAT** **(Hedgehog acyltransferase)**	a transmembrane acyltransfrase localised at ER	embryonic lethal	holoprosencephaly, acrania (malformation of the skull) and agnathia (no jaw)	Dennis et al., 2012	Holoprosencephaly and craniofacial defects	Dennis et al., 2012
	**Dispatched**	membrane receptor	DispA mutants die at e9.5; DispB mutants are viable	smaller body size, neural patterning defect, holopocensephaly	Ma et al., 2002	holoprosencephaly-like microform	Roessler et al., 2009; Kantarci et al., 2010
	**Scube2** **(Signal Peptide, CUB Domain and EGF Like Domain Containing 2)**	secreted and membrane-tethered	viable	Impairment of endochondral bone formation	Lin et al., 2015	Reduced Scube2 expression level is associated with colorectal cancer	Song et al., 2015
	**Chmp1a** **(Charged Multivesicular body Protein 1a)**	endosomal protein (ESCRT-III)	perinatal lethal	reduced body size, microcephaly, basal ganglia and cerebellar hypoplasia	Coulter et al., 2018	pontocerebellar hypoplasia (pons and cerebellar hypoplasia)	Mochida et al., 2012
	**Ext1** **(Exostosin Glycosyltransferase 1)**	Glycosyltransferase, producing heparan sulfate	KO: die at gastrulation hypomorphic mutant: die e14.5	reduced skeleton size with fused vertebrae, shortened limbs	Koziel et al., 2004	exostosis, chondrosarcoma	Ahn et al., 1995; Hecht et al., 1997; Philippe et al., 1997; Raskind et al., 1998; Faiyaz-Ul-Haque et al., 2004; Heinritz et al., 2009
	**NPC1** **(Niemann Pick type C1)**	cholesterol transporter with transmembrane domains	viable	progressive neuronal loss in the cerebellum	Loftus et al., 1997; Canterini et al., 2017; Formichi et al., 2018	premature death; lumsiness, learning difficulties, ataxia, dysphagia,vertical gaze palsy	Garver et al., 2007
	**DHCR7** **(7-dehydrocholesterol reductase)**	enzyme	neonatal lethal (within 24 hours after birth)	lung hypoplasia	Yu et al., 2004; Yu et al., 2005	Smith-Lemli-Opitz syndrome (SLOS); congenital malformations, intellectual disability, epileptiform activity and autism spectrum disorder	Patrono et al., 2002; Cooper et al., 2003; Blassberg et al., 2016
	**EHD1** **(EPS15-Homology Domain-containing protein 1)**	ATP and membrane binding protein; endocytic recycling	prenatal lethal in B6 background; normal in 129/SvEv or SwissWebster background	failure of neural tube closure, axial turning and patterning of the neural tube; in the viable cases, ocular lens development, muscle development and spermatogenesis affected	Rainey et al., 2010; Arya et al., 2015; Bhattacharyya et al., 2016	No disease-causing mutations identified	
	**GPC3** **(Glypican-3)**	Heparan sulfate proteoglycan	can be born, but die before weaning	pre/postnatal overgrowth	Cano-Gauci et al., 1999	Simpson-Golabi-Behmel overgrowth syndrome (SGBS), pre-and postnatal overgrowth with visceral and skeletal anomalies	Neri et al., 1998; Capurro et al., 2008
**membrane receptors** **and their associated proteins**	**Ptch1** **(Patched1)**	12-span transmembrane protein	die by e10.5	the neural tube fails to close completely. Overgrown head folds, hindbrain, and spinal cord	Marigo et al., 1996; Goodrich et al., 1997	basal cell nevus syndrome (BCNS), Gorlin syndrome, medulloblastoma	Goodrich et al., 1997
	**Ptch2** **(Patched2)**	12-span transmembrane protein	viable, fertile	KO; alopecia and skin lesions mutant; defect in opic cup morphogenesis, and optic fissure and stalk formation	Motoyama et al., 1998b; Nieuwenhuis et al., 2006	basal cell nevus syndrome (BCNS), Gorlin syndrome, medulloblastoma	Fujii et al., 2013
	**Smo** **(Smoothened)**	G-protein coupled receptor	die by e9.5	fail to turn, arresting at somite stages with a small, linear heart tube, an open gut and cyclopia; similar to the phenotypes in Shh/Ihh double KO	Zhang et al., 2001a	active mutations: basal cell carcinoma, Curry-Jones Syndrome	Xie et al., 1998; Twigg et al., 2016
	**Hhip** **(Hedgehog-interacting protein 1)**	membrane protein	neonatal lethal	lung and endochondral skeleton development is affected	Chuang and McMahon, 1999; Chuang et al., 2003	decreased expression of Hhip found in chronic obstructive pulmonary disease (COPD) patients	Zhou et al., 2012
	**Gas1** **(Growth-arrest-specific 1)**	GPI-anchored cell surface protein	die within 3 days after birth	eye, cerebellar and limb deficiencies; midfacial hypoplasia, premaxillary incisor fusion, and cleft palate	Lee et al., 2001; Liu et al., 2001; Allen et al., 2007; Seppala et al., 2007	Holoprosencephaly	Ribeiro et al., 2010; Pineda-Alvarez et al., 2012
	**Cdo/Cdon** **(Cell-adhesion molecule-related, down-regulated by oncogenes)**	a membrane protein of Immunoglobulin superfamily	perinatal lethal; 60% die within 21 days after birth	mild holoprosencephaly; midline structures lost	Cole and Krauss, 2003; Tenzen et al., 2006; Zhang et al., 2006; Allen et al., 2007	Holoprosencephaly	Bae et al., 2011
	**Boc** **(Brother of Cdo)**	a membrane protein of Immunoglobulin superfamily, similar to Cdo	viable	misguidance of commissural axons towards the floor plate.	Okada et al., 2006	modifying the expressivity of holoprosencephaly	Hong et al., 2017
	**LRP2/megalin/GP330** **(Low density lipoprotein-related protein 2)**	single-span receptor	perinatal lethal; 60% die within 21 days after birth	defective forebrain develoipment	Willnow et al., 1996; Christ et al., 2012; Baardman et al., 2016	Donnai-Barrow syndrome; HPE-like phenotypes	Kantarci et al., 2007
	**casein kinase 1a (CK1α)**	serine/threonine kinase	die before e6.5	unknown	Elyada et al., 2011	overexpressed in colorectal cancer	Richter et al., 2018
	**G protein-coupled receptor kinase 2 (GRK2)**	serine/threonine kinase	die at e9–12	marked cardiac abnormalities	Jaber et al., 1996; Penela et al., 2010	GRK2 is upregulated in granulosa cell tumours and in thyroid carcinoma	Penela et al., 2010
	**β-Arrestin2**	GPCR modulator	viable	sensitivity to pain change; reduced generation of amyloid-b peptide	Bohn et al., 1999; Thathiah et al., 2013	reduced level of amyloid-β peptide, hepatocellular carcinoma	Thathiah et al., 2013; Sun et al., 2016
	**Kif3a**	kinesin-like protein	die at e10.5	randomization of laterality in heart looping (sinus inversus)	Takeda et al., 1999	maybe involved in Usher's syndrome (photoreceptor degeneration), but no case reports have been given	Lefevre et al., 2008; Sipe and Lu, 2011
**signal mediators**	**Costal2/Kif7**	Kinesin-4 family, with an N-terminal motor domain	die at the end of gestation	preaxial polydactyly, exencephaly, and microphthalmia	Endoh-Yamagami et al., 2009; Liem et al., 2009; Hsu et al., 2011; He et al., 2014a	fetal hydrolethalus and acrocallosal syndromes	Putoux et al., 2011
	**SuFu** **(Suppressor of Fused)**	scaffold protein	die at e9.5	ventralised neural tube (similar phenotypes to those of Ptch1 mutant)	Svard et al., 2006; Chen et al.,. 2009	Gorlin syndrome, JBTS32 congenital ataxia, cerebellar vermis hypoplasia, cranio-facial dysmorphisms, polydactyly.	Svard et al., 2006; De Mori et al., 2017
	**Spop** **(Speckle-type POZ protein)**	E3 ubiquitin ligase adaptor	neonatal lethal	perturbation of skeletal development	Cai and Liu, 2016	serous tumors, prostate cancer	Zapata et al., 2001: Le Gallo et al., 2012; Zuhlke et al., 2014
**transcption factors**	**Gli1**	transcription factor (Krüppel-like zinc finger)	viable and fertile	no apparent phenotype	Park et al., 2000	Ellis–van Creveld syndrome (short limbs and ribs, postaxialpolydactyly, teeth and nail defects)	Peng et al, 2013; Palencia-Campos et al., 2017
	**Gli2**	transcription factor (Krüppel-like zinc finger)	die at birth	foregut defects, stenosis of the oesophagus and trachea, hypoplasia and lobulation defects of the lung. No floor plate in the neural tube	Mo et al., 1997; Ding et al., 1998; Motoyama et al., 1998b	substitute mutations: pituitary anomalies and holoprosencephaly-like features	Roessler et al., 2003
	**Gli3**	transcription factor (Krüppel-like zinc finger)	viable and fertile	enlarged maxillary arch, a reduced external nasal process, poorly developed eyes, misplaced ears, anomalous numbers of mystacial and supra-orbital hair	Hui and Joyner, 1993; Litingtung and Chiang, 2000	Greig cephalopolysyndactyly syndrome (GCPS), postaxial polydactyly	Pettigrew et al., 1991; Wild et al., 1997

### Expression and Importance of Shh in Neural Tube and Limb Bud Development

Hh genes are expressed in a variety of tissues throughout developmental and postnatal stages (Petrova and Joyner, 2014), and play critical roles in the development and homeostasis of these tissues. Among the three vertebrate Hh genes, Shh is highly expressed starting at early developmental stages and shows strong activity; therefore, it has been analyzed extensively. Shh expression is initially detected in the axial mesoderm in the head and caudal areas at embryonic day 7.5 (e7.5) (Echelard et al., 1993; Marti et al., 1995; Blaess et al., 2014). At e8.5, Shh expression is detected in the neural tube (i.e., the embryonic CNS). Shh expression in the neural tube is restricted to the ventral region. At the trunk level, Shh is expressed in the floor plate domain, which is located at the ventral-most part of the neural tube. In addition to its expression in the nervous system and the axial mesoderm, Shh is expressed in the zone of polarizing activity (ZPA) of the fore- and hind-limb starting at e9.5 (Echelard et al., 1993). This expression pattern is maintained until late developmental stages (Echelard et al., 1993).

One important characteristic of Shh is that it acts as a morphogen. Morphogens are a group of signaling (secretory) molecules that determine different cell fates in a concentration-dependent manner (Wolpert, 1969; Cohen et al., 2013; Matise, 2013; Briscoe and Small, 2015; Tickle and Towers, 2017). Shh is expressed in the floor plate in the neural tube, and its levels reach a concentration gradient along the dorsal-ventral axis, assigning different neural identities in a concentration-dependent manner (Alaynick et al., 2011). This is experimentally proven by incubating neural progenitor cells with different concentrations of Shh, which results in the production of different neural subtypes after a certain time period (Dessaud et al., 2007; Dessaud et al., 2010). However, the concentration of Shh in the neural tube changes dynamically over time during embryogenesis, and the distribution of the Shh protein does not correspond to intracellular Shh activity or Gli activity (Balaskas et al., 2012). Cells responsive to Shh show a rapid increase in activity followed by a gradual decrease. Multiple mechanisms for this negative feedback (termed "temporal adaptation") (Dessaud et al., 2007; Cohen et al., 2015) have been proposed. One mechanism involves the expression of *Ptch* (Dessaud et al., 2007); the *Ptch* gene is induced by Shh, and the accumulation of Ptch in the ciliary membrane blocks the Shh signal (Dessaud et al., 2007). Moreover, Gli2 transcription in the neural tube gradually decreases during development, and this decrease downregulates the Shh signal transduction over time (Cohen et al., 2015). In addition, the new regulator GPR17, which plays an essential role in this temporal adaptation, was recently isolated (Yatsuzuka et al., 2019).

In the limb bud, a classical experiment shows that transplantation of ZPA into the anterior side of the limb bud induces an extra number of fingers, which is called "polydactyly" (Tickle and Towers, 2017). Therefore, the regulation of Shh activity in the limb bud is critical for proper digit formation.

Shh is expressed in the CNS and limb bud starting at early stages, and it plays essential roles in the differentiation and construction of these tissues. Mice deficient in the Shh gene have holoprosencephaly (smaller head) with cyclopia (single eye), and lack ventral cell types within the neural tube of the spinal cord and in most of the ribs. Consequently, knockout mice usually die by e10.5 (Chiang et al., 1996; Bulgakov et al., 2004).

*Shh* is also expressed in the lung, which is an endoderm-derived organ, and Shh is expressed in the distal epithelium at least from e10.5 to e16.5. Shh is detected in epithelial cells both in peripheral and conducting airways (Miller et al., 2001). Analysis of *Shh* knockout mice with a mild phenotype shows defects in foregut development and lung branching morphogenesis (Litingtung et al., 1998; Pepicelli et al., 1998).

### Adult Neural Stem Cells and Shh

Although Shh plays essential roles in various developmental stages, it is also expressed and plays important roles at postnatal stages (Alvarez-Buylla and Ihrie, 2014; Petrova and Joyner, 2014), particularly in brain neural stem cells. In the adult brain, two regions undergo continuous neurogenesis: the ventricular subventricular zone (V-SVZ) and the subgranular zone (SGZ) of the dentate gyrus (Ahn and Joyner, 2005; Alvarez-Buylla and Ihrie, 2014). After these regions are established (Ahn and Joyner, 2005), both quiescent and transit-amplifying neural stem cells respond to Shh and start to expand (Ahn and Joyner, 2005; Alvarez-Buylla and Ihrie, 2014). Shh is required for the maintenance of stem cells and migrating newborn neurons (Balordi and Fishell, 2007a). Analysis of tamoxifen-inducible *Smo* mutants shows that both proliferation and neurogenesis of neural stem cells are compromised (Balordi and Fishell, 2007b). Therefore, Shh is required for the maintenance, proliferation, differentiation, and migration of adult neural stem cells.

### Ihh and Dhh

*Ihh* is mainly expressed in the developing skeletal system. The *Ihh* mRNA can be detected in cartilage at e11.5, and later in prehypertrophic chondrocytes (Bitgood and McMahon, 1995; St-Jacques et al., 1999). In developing chondrocytes, *parathyroid hormone-related peptide* (*PTHrP*) is one of the target genes of *Ihh*. PTHrP in turn antagonizes Ihh and sequesters the Ihh signal, and thereby proceeds differentiation.

Half of the *Ihh* knockout mice die between e10.5 and e12.5, probably because of circulatory abnormalities. The rest of the mutant mice remain alive until the end of gestation, but die at birth from respiratory failure. The mutant embryos are smaller in size and have short forelimbs with reduced chondrocyte proliferation (St-Jacques et al., 1999; Karp et al., 2000; Levi et al., 2011). *PTHrP* expression is downregulated in *Ihh* knockout mice (Karp et al., 2000). However, constitutive expression of PTHrP in the *Ihh* knockout background cannot rescue all *Ihh* knockout phenotypes, suggesting the existence of a *PTHrP*-independent pathway involved in the short-limb phenotype or decreased chondrocyte proliferation.

Although *Ihh* knockout mice are embryonic-lethal, the postnatal function of Ihh was elucidated by conditional gene disruption in a tamoxifen-inducible system (Zhou et al., 2014). In these conditional knockout mice, surgically induced osteoarthritis (OA) was attenuated, whereas wild-type mice showed OA progression (Zhou et al., 2014). This result provides possible novel therapeutic strategies to prevent OA progression.

In the mouse, *Dhh* is expressed in Sertoli cells immediately after sex determination at e11.5 (Bitgood et al., 1996) and in Schwann cells, which are a component of the peripheral nervous system, at e14.5 (Bitgood and McMahon, 1995). *Dhh* expression lasts until the postnatal stages (Bitgood and McMahon, 1995; Bitgood et al., 1996). *Dhh*-deficient mice are viable, and show sex-specific phenotypes. Female knockout mice do not exhibit an obvious phenotype and are fertile, whereas mutant male mice show defects in spermatogenesis and are therefore infertile (Bitgood et al., 1996; Clark et al., 2000). In the adult testis, *Dhh* is expressed in Sertoli cells, and *Dhh* knockout mice lack *Ptch1* expression in neighboring Leydig cells, suggesting that Leydig cells are the target of Dhh (Bitgood et al., 1996). In the peripheral nervous system, detailed analysis of *Dhh* mutant mice showed a thin perineurium and abnormal formation of perineural tight junctions (Parmantier et al., 1999). These findings suggest that Dhh is also important for the development of peripheral nerve sheaths.

Taken together, these studies indicate that Hh signaling is critical not only during embryogenesis, but also for postnatal biological processes.

### Essential Functions of Genes Involved in the Hh Secretion Process

The acyltransferase HHAT is essential for embryonic development (Dennis et al., 2012). Deficiency of HHAT function perturbs long-range Hh transport in tissues, and affects the activity of other signaling molecules of FGF and BMP as a secondary effect, which results in holoprosencephaly and craniofacial defects (Dennis et al., 2012; Konitsiotis et al., 2015).

Chmp1a is an ESCRT-III component that mediates Shh secretion through the endosome. Shh secretion is impaired in *Chmp1a* knockout mice, and mutant mice show a small body size and microcephaly (small brain), and die soon after birth (Coulter et al., 2018). This phenotype can be reversed through crossing with a *Ptch1* heterozygote. Ptch *per se* antagonizes the Hh signal by blocking Smo activity, suggesting that Hh signaling is activated in the *Ptch* mutant (Goodrich et al., 1997). The reversal of the phenotype by the heterozygotic reduction of *Ptch* expression under the *Chmp1a*-deficient background suggests that the phenotypes caused by single *Chmp1a* gene knockout are caused by decreased Hh function (Coulter et al., 2018).

The ESCRT-II subunit Vps25 (Handschuh et al., 2014) does not directly affect Shh secretion. However, degradation of the FGF receptor is perturbed in Vps25 hypomorphic mutants. This increases FGF activity and accelerates the feedback loop between FGF and Shh involved in limb bud development. Deficiency of the Vps25 gene therefore represents aberrant upregulation of Shh, and results in the polydactyly phenotype (Handschuh et al., 2014).

Two proteins of Disp and Scube2 are related to the Hh protein releasing process. *DispA* is detected from e7.5 throughout the embryo stage (Ma et al., 2002), and *DispA* mutant embryos die at e9.5 with defects in head structure and abnormal neural patterning (Ma et al., 2002). In mutant mice, the Hh modification is intact, but the secretion process is perturbed (Ma et al., 2002). Scube2 encodes a secreted and membrane-tethered protein with complement C1r/C1s, Uegf, Bmp1 (CUB)- (Bork and Beckmann, 1993) and EGF-like domains, and plays essential roles in chondrocyte differentiation and cell proliferation (Lin et al., 2015). Detailed analysis revealed that Scube2 is a strong promoter of Ihh activity (Lin et al., 2015).

After Hh proteins are released from producing cells, their stability and tissue distribution depend on the extracellular environment. For instance, vitronectin is an extracellular matrix protein that acts synergistically with Shh during motor neuron differentiation (Pons and Marti, 2000). By contrast, the heparan sulfate (HS) chain, synthesized by the glycosyltransferase exostosin1 (Ext1), restricts the spread of Hh (Koziel et al., 2004; Callejo et al., 2006). Ext1 acts in the Golgi apparatus and catalyzes the elongating reaction of the HS tetrasaccharide chain, thereby contributing to the production of HS proteoglycan (HSPG) (Koziel et al., 2004). HSPG binds to Ihh and restricts Ihh signaling in chondrocytes. In *Ext1* hypomorphic mutants (mutants in which the Ext1 expression is significantly reduced), HS synthesis is suppressed and hypertrophic chondrocyte differentiation is delayed, presumably because Ihh is spread in the tissue (Koziel et al., 2004). In a similar process, deficiency of the Ext2 gene results in the reduction of HS synthesis and early embryonic lethality (i.e., embryos die around e6.5) (Stickens et al., 2005).

One of the glypican family proteins, glypican-3 (GPC-3), is expressed on the surface of Hh-receiving cells and directly binds to Hh proteins (Capurro et al., 2008), thereby suppressing Hh signaling (Capurro et al., 2008). This activity involves endocytosis and degradation of the GPC-3-Hh complex, and low-density lipoprotein receptor-related protein-1 plays an essential role in this process (Capurro et al., 2012).

The interaction between GPC-3 and Hh competes with the Ptch-Hh interaction. Therefore, the phenotypes of *GPC-3* mutant mice are related to excessive Hh signaling, and are characterized by embryonic overgrowth (heavier embryos) with expanded ventral identity in the spinal cord, and renal and lung anomalies (Cano-Gauci et al., 1999; Chiao et al., 2002).; Capurro et al., 2008)

### Membrane Receptors

The main function of Ptch1 (Goodrich et al., 1997) and Ptch2 (Alfaro et al., 2014; Zhulyn et al., 2015) in Hh signaling is to inhibit Smo activity, thereby maintaining the quiescence of the Hh signaling pathway. Knockout of the *Ptch1* gene causes constitutive activation of Smo and its downstream Hh pathway, and the phenotype is reminiscent of that caused by overactivation of the Hh pathway. For instance, *Ptch1* knockout mice, which die by e10.5, show a ventralized neural tube (Goodrich et al., 1997). Ptch2 plays redundant roles during embryogenesis (Motoyama et al., 1998b; Zhulyn et al., 2015), and the phenotypes of *Ptch2* mutants are manifested at postnatal stages; the mutation causes alopecia (hair loss) and epidermal hyperplasia (Nieuwenhuis et al., 2006).

Studies of another membrane protein, Hhip, show that *Hhip* knockout mice die at neonatal stages because of lung and endochondral skeleton defects (Chuang et al., 2003). Conversely, transgenic mice in which Hhip is overexpressed in cartilage show a shortened skeleton, which is similar to the phenotype of *Ihh* knockout mice (Chuang and McMahon, 1999), suggesting that Hhip is a negative regulator of the Hh signal.

During the translocation of Smo into primary cilia, phosphorylation of the serine residues at the carboxyl-terminal region is essential for its full function. Although CK1α and GRK2 are essential for the phosphorylation and translocation of Smo into cilia, they have a wide variety of substrate proteins, namely, Smo is not the only substrate for these kinases. Therefore, the phenotypes associated with mutations of each gene are more severe or distinct from the ones expected from the deficiency of Hh signaling molecules (Jaber et al., 1996; Elyada et al., 2011).

β-Arrestin 2 also has a variety of partner proteins. In zebrafish, mutation of the *Smo* gene and knockdown of *β-arrestin 2* produce similar phenotypes characterized by defects in muscle and ventral neural tube differentiation. Moreover, the phenotypes caused by the *Smo* mutation can be rescued by overexpression of β-arrestin 2, suggesting that there are strong epistatic connections between *Smo* and *β-arrestin 2* (Wilbanks et al., 2004). However, *β-arrestin 2* mutant mice show different phenotypes involving changes in pain sensitivity (Bohn et al., 1999) and the generation of amyloid β-peptide, which is involved in Alzheimer's disease (AD) (Thathiah et al., 2013), in postnatal stages. Therefore, the dependency on β-arrestin 2 during development may be species-specific, and is partly redundant with other genes in the mouse.

When Smo is translocated into cilia, the endocytic recycling regulatory protein EPS15-homology domain-containing protein 1 (EHD1) (Rainey et al., 2010; Arya et al., 2015; Bhattacharyya et al., 2016) directly interacts with Smo, and is co-trafficked into primary cilia. *EHD1*-null cells show alterations in the shape of cilia, and the embryonic phenotype is the ventralized neural tube, which is caused by hyperactivation of Hh signaling (Bhattacharyya et al., 2016). This suggests that EHD1 affects the localization or the stability of the Smo protein.

Gas1, Cdo, and Boc are expressed in the dorsal part of the neural tube and in the anterior two-thirds of the forelimb bud in e10.5 mouse embryos, which is complementary to the expression of Shh (Allen et al., 2011). However, overexpression of each of these factors in the neural tube induces the ventralization of the neural tube, suggesting that these factors support Shh signaling (Allen et al., 2011). Cdo and Boc have partially redundant roles in neural tube dorso-ventral pattern formation and digit specification during limb bud development. Cdo/Boc compound mutants show a markedly defective structure, whereas the phenotypes are subtle in each single mutant. By contrast, the Gas1 mutant on its own has a strong phenotype in these tissues (Allen et al., 2011). Triple mutants deficient in the three receptors Gas1, Cdo, and Boc display severe forebrain and cardiovascular defects at e8.5, and later holoprosencephaly, cyclopia, and neural tube pattern defects (Allen et al., 2011), which resemble the Shh/Ihh compound or Smo knockout phenotypes (Zhang et al., 2001b). The mechanisms underlying the coordination of Hh signal reception between Gas1, Cdo, and Boc and the conventional receptor system consisting of *Ptch/Smo* need to be addressed in the future.

### Intracellular Signaling Mediators and Gli Proteins

Among mediators of the Hh signaling pathway, SuFu and its modulator Kif7 play critical roles in tissue morphogenesis, and the respective mutants therefore exhibit severe phenotypes.

*SuFu* mutant mice exhibit a severely ventralized pattern in the neural tube, suggesting that SuFu is a negative regulator of Hh signaling (Svard et al., 2006; Chen et al., 2009).

Kif7, a kinesin-4 family motor protein, localizes to the distal tip of primary cilia *in vivo* and determines the length of cilia (He et al., 2014a). The Kif7 mutant is characterized by long and twisted cilia (He et al., 2014a).

Kif7 binds to Gli2/3, and Gli activity is aberrantly upregulated in *Kif7* mutant cells (Cheung et al., 2009). In addition, ventral domains in the neural tube are expanded and polydactyly is observed in the mutant (Endoh-Yamagami et al., 2009; Liem et al., 2009), suggesting that Kif7 acts as a negative regulator of Hh signaling. In addition, Kif7 mutation partially rescues the phenotype caused by *Smo* knockout, confirming that Kif7 acts as a negative effector in the Hh pathway downstream of *Smo* (Liem et al., 2009). However, the effect of combined *Kif7* knockout and *Ptch1* mutation on rescuing the phenotype indicates that Kif7 can also promote the activity of the Shh pathway (Liem et al., 2009).

Kif7 functions both as a positive and a negative factor in chondrocyte differentiation also in the growth plate (Hsu et al., 2011). Kif7 plays a positive role for the Hh activity by excluding the Gli-SuFu complex from primary cilia, thereby limiting the repressive effect of Gli. However, in the absence of *SuFu*, Kif7 inhibits Gli transcriptional activity (Hsu et al., 2011). The dual role of Kif7 may be dependent on other partner proteins forming a complex with SuFu and Kif7.

Spop regulates the stability of the Gli2 and Gli3 proteins by ubiquitinating at their carboxyl-terminal domains (Wang et al., 2010). However, the phenotypes in the Spop mutants are more complex than expected. Single *Spop* deficiency does not severely affect dorsal-ventral pattern formation in the neural tube (Cai and Liu, 2017). On the other hand, *Spop* and *Gli2* compound mutants rescue the loss of ventral neural identity caused by the Gli2 single mutant, suggesting a negative role of Spop in Gli activity (Cai and Liu, 2017). However, during skeletal development, *Spop* deficiency results in the accumulation of Gli3R, and causes defects in chondrocyte and osteoblast differentiation (Cai and Liu, 2016). Therefore in this context, Spop acts as a positive regulator of Ihh signaling/skeletal development. These findings indicate that the activity of Spop in the Hh signaling pathway is context-dependent.

At the transcriptional level, Gli genes play partially overlapping roles in mediating Shh intracellular signaling (Bai and Joyner, 2001; Lei et al., 2004). However, the three genes have different requirements. Gli1-deficient mice do not show apparent phenotypes and are viable and fertile (Park et al., 2000), whereas Gli2 knockout mice die at birth (Motoyama et al., 1998a). Gli3 mutants are viable and exhibit tumorigenic phenotypes with craniofacial anomalies (Hui and Joyner, 1993; Veistinen et al., 2012). Moreover, Gli2 and Gli3 have partially redundant functions in limb bud development (Mo et al., 1997). The different requirements of Gli proteins may be related to spatial differences in the expression of the three Gli proteins.

### Fatty Acids, Lipids, and Processing of Hh Molecules

Cholesterol biosynthetic enzymes play indirect but important roles in Hh signaling. Cholesterol and fatty acids are synthesized from acetyl-CoA through several steps. The enzyme 7-dehydrocholesterol reductase (DHCR7) converts 7-dehydrocholesterol to cholesterol in the last step of cholesterol synthesis (Yu et al., 2004; Blassberg et al., 2016). Mice deficient in the *DHCR7* gene die immediately after birth from lung hypoplasia (Yu et al., 2004). At the molecular level, full activation of Smo requires cholesterol binding at the extracellular cysteine-rich domain (Nedelcu et al., 2013; Luchetti et al., 2016), which induces a conformational change in Smo to its active state (Huang et al., 2016; Myers et al., 2017; Xiao et al., 2017).

The NPC1/2 proteins are also critical for Hh modification. NPC1/2 transport cholesterol from the endosome to the ER/Golgi (Urano et al., 2008), and this transport is blocked in *NPC1/2*-deficient mice. This results in the accumulation of cholesterol in the lysosome, which prevents cholesterol modification of Hh proteins as well as Ptch and Smo (Orth and Bellosta, 2012) and the production of active proteins. NPC1/2-deficient mice are characterized by increased cholesterol levels and progressive neuronal loss in the cerebellum (Loftus et al., 1997; Canterini et al., 2017; Formichi et al., 2018). In addition, shortened primary cilia are detected in *NPC1* mutant cells (Formichi et al., 2018), suggesting that NPC affects the production of sterols in cilia, and Hh signal transduction is perturbed not only at the secretion level but also at the receptor level.

### Mutations in Hh Target Genes

Several target genes are induced by Hh signals. In the spinal cord, the transcription factors that determine neural identity are induced by Shh in a concentration-dependent manner (Dessaud et al., 2008) and form regulatory networks (Briscoe and Small, 2015). The transcription factors FoxA2, Olig2, and Nkx2.2 have Gli-binding sites in their regulatory domains (Vokes et al., 2007; Kutejova et al., 2016). Olig2 and Nkx2.2, which are basic helix-loop-helix and homeobox transcription factors, respectively, are induced by Shh and repress each other (Kutejova et al., 2016). In addition, Nkx2.2 (expressed in the ventral p3 domain) and Pax6 (expressed in the intermediate domains) are mutually repressive (Briscoe et al., 2000). Knockout of each of these components perturbs the transcriptional network and alters pattern formation (Balaskas et al., 2012).

In limb development, Shh induces a set of genes distinct from those involved in neural development (Vokes et al., 2008; Lewandowski et al., 2015). For instance, the transcription factor Blimp1, which is expressed in the posterior forelimb, is a direct target gene of Shh and is essential for the maintenance of ZPA and other mesodermal tissues in mice (Robertson et al., 2007). Gremlin and Hand2, which encode a BMP antagonist and a transcription factor, respectively, are expressed in a region distinct from that of Shh. Nevertheless, they have Gli-binding sites in their regulatory regions and are directly regulated by active Gli (Vokes et al., 2008). As in the neural tube, Shh acts in a long-range and concentration-dependent manner in the limb bud, and target gene expression is regulated both spatially and temporally.

In the Ihh pathway, because *PTHrP* is a target gene of Ihh signaling, disruption of *PTHrP* or its receptor *PTH/PTHrP-R* results in skeletal dysplasia, premature maturation of chondrocytes, and excessive bone formation, and causes a lethal phenotype (Karaplis et al., 1994; Lanske et al., 1996). Overall, the critical roles of Hh molecules are at least partly reflected in the downstream factors and their transcriptional networks.

### Non-Canonical Hh Pathways and Their Biological Roles

Although the pathway composed of Ptch, Smo, Gli, and their associated factors plays a major role in Hh function, other pathways that bypass this canonical pathway have been analyzed. Shh functions in axon guidance in commissural axons (Charron et al., 2003; Hammond et al., 2009; Aviles et al., 2013) in collaboration with the long-range diffusible chemoattractant netrin (Kennedy et al., 1994; Charron et al., 2003). Notably, Shh-dependent axon guidance is not mediated by Ptch, but Boc (not Cdo) acts as a receptor for Shh (Okada et al., 2006). *Boc* knockout mice show misplaced axons invading into the motor column (Okada et al., 2006).

Shh acts as an attractant before commissural axons cross the midline, whereas it acts as a repellent for post-crossing axons. This is related to changes in the protein composition of the growth cone. Accumulation of the adaptor protein 14-3-3 in a time-dependent manner results in repulsive axons (Yam et al., 2012). Because of this change in responsiveness, immediately after crossing the midline the axons are redirected perpendicularly to the rostral direction, where Shh concentration is low (Bourikas et al., 2005). Recently it has been shown that Boc is internalized into early endosomes and this endocytosis is required for the growth-cone turning of neurons (Ferent et al., 2019). Boc is also enriched in ipsilateral retinal ganglion cells (RGCs), and Shh, expressed at the midline, acts as a repellent for the RGCs, which thereby regulates the segregation of ipsilateral and contralateral neurons (Fabre et al., 2010).

In chick, *Hhip* is transiently expressed in the commissural axon at the time when axons turn into the longitudinal axis (Bourikas et al., 2005). However, this mechanism is not detected in mice, suggesting that the mechanism is species-specific.

Src family kinases (SFKs) are downstream Boc signaling effectors (Yam et al., 2009). While SFK activation is required for axonal guidance, it is not involved in Gli-mediated transcription. In Shh-mediated chemotaxis, cilia are not essential for pathway activation, suggesting that the role of the Shh pathway in chemotaxis and axon guidance is mediated by other factors than Ptch-Smo-Gli (Bijlsma et al., 2012). The extension of the axon correlates with the Wnt signal, supporting a mechanistic difference in the Shh pathway from that mediated by the Ptch-Smo-Gli axis (Aviles et al., 2013).

## Genetic Diseases Caused by Abnormalities in Hedgehog-Related Genes

Mutations or deletions in genomic regions, either in coding or non-coding regions, cause malfunctions of the translated proteins or alterations in gene expression levels, resulting in genetic disorders. The phenotypic penetrance caused is variable in humans compared with that resulting from gene knockout or disease models in mice, as a pure genetic background and rearing under standardized conditions confer sensitivity to genetic mutations. In human mutations, complete removal of genes is rare, as genomic mutations normally encode proteins with incomplete or submaximal levels of activity. Nevertheless, mutations of the indicated Hh-related genes result in severe hereditary disorders in humans, and in most cases, the effects are similar to those in knockout mice (overall symptoms based on the mutations of the Hh-related genes are summarized in **Table 1**, and the detailed symptoms are shown in **Table 2**).

**Table 2:** Detailed symptoms in the Hh-related disease. ND, not determined.

Disease name	Symptoms	Major causal genes (Hh-related)	References
**skeletal defects**			
Holoprosencephaly (HPE)	abnormal brain and facial structure; midfacial clefts such as cleft lip and palate, cyclopia (single eye)	Shh, HHAT, Disp, Cdo, Gas1, Gli2	Roessler et al., 1997; Heussler et al., 2002; Roessler et al., 2003; Roessler et al., 2009; Kantarci et al., 2010; Bae et al., 2011; Pineda-Alvarez et al., 2012 Dennis et al., 2012;
Greig cephalopolysyndactyly syndrome (GCPS)	abnormal development of the limbs, head, and face	Gli3	Pettigrew et al., 1991; Wild et al., 1997
Brachydactyly	short fingers	Ihh	Byrnes et al., 2009; Gao et al., 2001
acrocapitofemoral dysplasia	short limbs, relatively large head and narrow thorax	Ihh	Hellemans et al., 2003
hereditary multiple exostoses (HME)	reduced skeletal size and multiple, cartilage-capped, accompanied with benign bone tumors (exostoses)	Ext1/2	Wuyts et al., 1998; Beltrami et al., 2016
Gorlin's syndrome	a high risk of tumorigenesis, especially skin cancer. Also develop noncancerous (benign) tumors of the jaw.	Ptch1	Fujii et al., 2013
Curry-Jones Syndrome	multisystem disorder; patchy skin lesions, polysyndactyly etc.	Smo (active mutations)	Zhou et al., 2012
Donnai-Barrow syndrome	HPE-like phenotypes	LRP2	Kantarci et al., 2007
Acrocallosal syndromes	brain abnormality (failure of the corpus callosum development), extra fingers and toes (polydactyly), distinctive facial features.	Kif7	Putoux et al., 2011
**ciliopathies**			
Joubert syndrome	eye abnormalities (such as retinal dystrophy), kidney disease, liver disease, extra fingers and toes,	Kif7, SuFu	Aguilar et al., 2012; De Mori et al., 2017
Meckel syndrome	sac-like protrusions (Occipital encephalocele) or no major prtion of the brain (anencephaly), severely cystic kidneys, and abnormal liver and skeleton	Kif7	Aguilar et al., 2012
tumors, cancers			
medulloblastoma (MB)	neuroectodermal tumor in the cerebellum	Ptch2, SuFu	Rusert et al., 2014; Millard and De Braganca, 2016
basal cell carcinoma (BCC)	skin cancer	Ptch1, Ptch2, SuFu	Schulman et al., 2016
basal cell nevus syndrome (BCNS)	skeletal abnormalities, jaw keratocysts, calcification of brain structures, carcinoma	Ptch1, Ptch2, SuFu	Johnson et al., 1996; Kimonis et al., 1997; Goodrich et al., 1997; De Mori et al., 2017;
**neurological disorders**			
Parkinson’s disease (PD)	motor deficiencies (slowness of movement, tremors, and postural instability)	ND (involvement of Hh signal has been suggested)	Gonzalez-Reyes et al., 2012
Alzheimer’s disease (AD)	deterioration of cognitive and memory functions	ND (involvement of Hh signal has been suggested)	Vorobyeva and Saunders, 2018

### Developmental Disorders

Among the three Hh genes, *Shh*, *Ihh*, and *Dhh*, mutations in *Shh* or its abnormal expression produces severe symptoms in humans, including abnormalities in the development of the nervous system, facial structure, and limbs.

Holoprosencephaly (HPE) is a common disorder of brain development with a prevalence of 1 in 250 embryos and in 16,000 live births (Roessler et al., 1997). In HPE, the cerebral hemisphere is not formed, and the patients develop an abnormal brain and facial structure, with frequent midfacial clefts such as cleft lip and palate (Roessler et al., 1997). Severe cases of HPE, which are rare, show cyclopia (single eye) (Sharma et al., 2014). This syndrome is not only associated with mutations in *Shh* (Roessler et al., 1997; Heussler et al., 2002), but also with those in downstream genes. To date, HHAT (Dennis et al., 2012), Disp (Roessler et al., 2009; Kantarci et al., 2010), Cdo (Bae et al., 2011), Gas1 (Pineda-Alvarez et al., 2012), and Gli2 (Roessler et al., 2003) have been reported as causal genes for HPE.

Greig cephalopolysyndactyly syndrome (GCPS or Greig syndrome), which is characterized by abnormal development of the limbs, head, and face, is considered a genetic developmental disorder. It shows an autosomal dominant inheritance pattern (Pettigrew et al., 1991; Wild et al., 1997), and Gli3 has been recognized as the causative gene for GCPS and its related disorder, Pallister-Hall syndrome (Kang et al., 1997).

In addition *Shh*-related genes, the genes encoding the transcription factors Zic2, Six3, and TGF-β induced factor homeobox 1 (TGIF 1) are considered major causative genes for HPE (Dubourg et al., 2004; Dubourg et al., 2016). Recent studies using next generation sequencing show that the FGF signaling-related genes *FGF8* and *FGF receptor 1* are also causal genes (Dubourg et al., 2016).

Mutation of the *Ihh* gene causes defects in skeletal development, and is associated with brachydactyly (short fingers) (Gao et al., 2001; Byrnes et al., 2009) and acrocapitofemoral dysplasia (short limbs, relatively large head, and narrow thorax) (Hellemans et al., 2003). In all reported cases, the mutations are located in the amino-terminal active fragment of Ihh (Byrnes et al., 2009).

Another Ihh-related growth defect is hereditary multiple exostoses (HME), which is characterized by reduced skeletal size and multiple, cartilage-capped bony outgrowths, accompanied by benign bone tumors (exostoses) in endochondral bones (Wuyts et al., 1998; Beltrami et al., 2016). HME and two other related bone disorders, exostosis and chondrosarcoma, are found in families that have mutations in the *Ext1/2* genes (Ahn et al., 1995; Hecht et al., 1997; Philippe et al., 1997; Raskind et al., 1998; Faiyaz-Ul-Haque et al., 2004; Heinritz et al., 2009). Ext1 is essential for HS synthesis and proper Ihh signal distribution (Koziel et al., 2004) in the mouse, and the symptoms of HME are reminiscent of the mutant phenotypes.

Mutations in *Dhh* are associated with congenital disorders. *Dhh* mutation was found in a patient with premature female genitalia and an immature uterus, regardless of the XY karyotype (Umehara et al., 2000). This patient also suffered from neuropathy (Umehara et al., 2000), which is reasonable considering that *Dhh* is expressed in the peripheral nervous system (Parmantier et al., 1999).

### Ciliopathies

Primary cilia act as a cellular "antenna" that receives chemical and mechanical signals from the extracellular environment to transduce them into the cells, and play important roles in development and homeostasis (Sasai and Briscoe, 2012; Wheway et al., 2018). Several transmembrane receptors related to Wnt, Notch, Hippo, and platelet-derived growth factor, as well as the receptors for Hh (i.e., Ptch, Gli; Figure 1) localize to cilia (Sasai and Briscoe, 2012; Malicki and Johnson, 2017; Wheway et al., 2018). Therefore, ciliary dysfunction abrogates signal transduction by these factors, resulting in a group of diseases termed ciliopathies.

The symptoms of ciliopathies are malformation of the body, including abnormal neural development (Waters and Beales, 2011; Guo et al., 2015) and *situs inversus* (major visceral organs are deployed with right and left sides reversed) (Pennekamp et al., 2015), during development, and retinal degeneration (Wheway et al., 2014; Bujakowska et al., 2017), kidney disease (Hildebrandt et al., 2009; Habbig and Liebau, 2015), and male infertility (Inaba and Mizuno, 2016) in the postnatal stages (Nigg and Raff, 2009; Waters and Beales, 2011; Shaheen et al., 2016). To date, more than 400 proteins have been identified and characterized, and many more unknown genes/proteins are expected to exist (Nigg and Raff, 2009; Goetz and Anderson, 2010; Reiter and Leroux, 2017).

Ciliary defects cause symptoms similar to those found in Hh signaling mutants such as polydactyly, encephalocele, neural tube defects, and cerebellar vermis hypoplasia (Huangfu et al., 2003; Sasai and Briscoe, 2012; Bangs and Anderson, 2017). Dysfunction of *Jbts17* (also known as *CPLANE1*), a gene mutated in Joubert syndrome, Meckel syndrome, and oral-facial-digital syndrome, causes ciliogenesis and ciliary trafficking defects, and results in decreased Shh target gene expression (Damerla et al., 2015; Toriyama et al., 2016). Disruption of the *Cep290* gene, which encodes a centrosomal protein, causes severe renal defects (Hynes et al., 2014), and treatment with purmorphamine, an agonist of Hh signaling (Briscoe, 2006), increases the number of ciliated cells. This suggests that Hh signaling is a potential therapeutic target in some ciliopathies. Similarly, mutations in *MKS1* (Weatherbee et al., 2009) and *MKS3* (Abdelhamed et al., 2013), which encode centrosome and orphan proteins, respectively, cause systemic defects related to ciliogenesis in association with alterations in Shh, and sometimes Wnt signaling.

Blockade of primary cilia normally perturbs the introduction of Hh into cells. However, lack of primary cilia caused by Kif3a mutation increases Hh activity in facial mesenchymal cells and results in craniofacial defects, hypertelorism (abnormal distance between the eyes), and frontonasal dysplasia (FND; developmental disorder with abnormal facial structure such as distant eyes and flat nose) (Wong et al., 2009; Brugmann et al., 2010).

Because the symptoms of ciliopathies are chronic, the patients are normally not easily treatable. Nevertheless, a number of trials have been made to cure these intractable diseases. For instance, gene therapy, where a viral infection of the functional ciliary genes into the body, has been shown to be successful to restore sensory input in the olfactory ciliopathy model mice (Green et al., 2018). In addition, by utilizing the method to make an organoid (a imitated three-dimensional organ differentiated *in vitro* from stem cells (Rossi et al., 2018)), a three-dimensional retina has been made from the iPS cells derived from the patients of retinitis pigmentosa, a type of ciliopathies. This method is useful because the progress of ciliopathies can be chased *in vitro*, and gives hints to find the strategies for the treatment (Deng et al., 2018). In addition, a microscopy-based automated system for small molecule screening was designed to search for new drugs that encourage ciliation. This system is expected to facilitate drug screening to find good chemicals to treat ciliopathies (Zhang et al., 2019).

### Medulloblastoma, Basal Cell Carcinoma, and Other Tumorigenic Disorders

Decreased Hh activity causes systemic tissue hypoplasia; however, overactive and persistent Hh signaling also leads to abnormalities in tissue homeostasis (Petrova and Joyner, 2014).

The representative phenotype induced by persistent activation of the Hh signal is cancer (Gupta et al., 2010). Two major types of cancer associated with Hh signaling have been extensively studied, namely, medulloblastoma (MB) and basal cell carcinoma (BCC) (Teglund and Toftgard, 2010).

MB is a primitive neuroectodermal tumor that most commonly arises from the cerebellum, and is normally diagnosed at a young age, especially in children younger than 10 years (de Robles et al., 2015). Approximately 200–500 cases are diagnosed in the United States every year (https://www.cancer.net/cancer-types/medulloblastoma-childhood/statistics). MB is divided into four types, Wnt-activated, Shh-activated, group 3, and group 4, according to diagnostic symptoms, genetics, and gene expression (Rusert et al., 2014; Millard and De Braganca, 2016). Approximately 30% of all cases, whether sporadic or genetic in nature, that are found in the cerebellar hemispheres (Rusert et al., 2014; Millard and De Braganca, 2016) are the Hh-activated type. In cases of germline mutations (genetic background), the *Ptch* and *SuFu* tumor suppressor genes tend to be abnormal (Yin et al., 2019).

BCC is a common skin cancer, accounting for >75% of all skin cancers (Zhang et al., 2001a). BCC is detected in 450–490 per 100,000 individuals in the UK (Epstein, 2008), and some BCC is associated with mutation of the *Ptch* gene (Gailani et al., 1996; Johnson et al., 1996). Analysis of biopsy samples from human BCC patients identified mutations in the *p53* and *Ptch1* genes in 30–50% and 20–30% of cases, respectively (Zhang et al., 2001a). Its related syndrome, basal cell nevus syndrome (BCNS), is characterized by skeletal abnormalities, jaw keratocysts, and calcification of brain structures in addition to carcinoma (Kimonis et al., 1997), and mutations in the *Ptch1*/*2* and *SuFu* genes have been found in BCNS patients (Johnson et al., 1996; Goodrich et al., 1997; Kimonis et al., 1997; De Mori et al., 2017).

Other types of cancer caused by the abnormal Hh expression include glioma (Gupta et al., 2010). Glioma is induced by the aberrant proliferation of glial cells both in the brain and in the spinal cord (Gupta et al., 2010).

A number of studies have shown that Hh, as well as Wnt, BMP, and Notch, pathway acts for the initiation step of glioma, namely gliomagenesis (Palma and Ruiz i Altaba, 2004; Laug et al., 2018). Importantly, human glioma-initiating cells (GICs; also known as glioma stem cells) undergo an indefinite self-renewal and are resistant to conventional anti-cancer drugs (Clement et al., 2007; Takezaki et al., 2011; Auffinger et al., 2015; Phi et al., 2018). On the other hand, it was demonstrated that overexpression of a C-terminal-truncated form of Gli2, exerted an antagonistic effect on the Gli activity and knocking-down of Gli1 and Gli2 expression prevented proliferation of GICs and their tumorigenesis (Clement et al., 2007; Takezaki et al., 2011). Therefore, it would be possible to cure the gliomagenesis by blocking the Shh signaling pathway, and as mentioned above, candidate chemicals are being tested for clinical applications (Pietrobono and Stecca, 2018).

As for therapy of the tumors, Hh antagonist could be used to block the signal. For instance, cyclopamine, which strongly blocks the Hh signal pathway, could have been a candidate chemical to ameliorate the symptoms (Chen et al., 2002). However, poor oral solubility and severe side effects in mice limited further clinical development of cyclopamine (Pietrobono and Stecca, 2018). Several other chemicals, which either bind to the Smo protein or inhibit its ciliary localization, have been developed and are currently under evaluation at preclinical or clinical trial stages (Pietrobono and Stecca, 2018). Among them, two Smo inhibitors, vismodegib and sonidegib, have already been approved by the US Food and Drug Administration (FDA) for treating the advanced BCC (Axelson et al., 2013; Migden et al., 2018; Pietrobono and Stecca, 2018; Niyaz et al., 2019), and clinical trials to apply these drugs for MB are ongoing as of October 2019 (Li et al., 2019). These drugs, especially sonidegib, can cross the blood-brain-barrier (Li et al., 2019) and have the advantage in the accessibility to tumors in the brain. In addition, statins, HMG-CoA reductase inhibitors, have been shown to synergize with Hh pathway inhibitors (e.g., vismodegib) in inhibiting the MB growth (Gordon et al., 2018). As cholesterol biosynthesis is required for Smo activity in the Hh signaling pathway, it is reasonable to decrease the cholesterol level to treat MB.

However, because Hh pathway is involved in many biological events, modification of Smo activity could cause a number of negative side effects. Therefore, a recent study proposed targeting a specific protein located in primary cilia (Bay et al., 2018). Knockdown of the *Arl13b* gene, which encodes a GTPase expressed in cilia, in MB cell lines suppresses only high level of Shh signaling and decreases cell proliferation. Loss of *Arl13b* in the *Ptch1-*null background abolishes the tumorigenic effect of loss of *Ptch1* (Goodrich et al., 1997), inhibiting MB formation in the developing cerebellum (Bay et al., 2018).

In the treatment of Hh-related cancers, GPCRs are considered effective targets because they are easily accessible from the cell exterior and could therefore be potential drug targets (He and Lu, 2015). Hh signal transduction is associated with the downregulation of PKA and decreased cAMP levels; therefore, chemicals that modify the levels of these molecules can be developed as an effective anti-cancer treatment. For instance, Gαs-type GPCRs upregulate intracellular cAMP and thus inhibit Hh signaling (He and Lu, 2015). For example, GPR161 overexpression changes the sensitivity of cells to Hh and is considered a potential drug target (Mukhopadhyay et al., 2013; He and Lu, 2015; Rao et al., 2016; Shimada et al., 2018; Pusapati et al., 2018a).

### Shh Signaling in Neurodegenerative Disorders

Parkinson's disease (PD) is one of the most common neurodegenerative disorders, with a prevalence of 1–2 in 1,000 people (Collaborators, G.B.D.P.s.D, 2018). PD is characterized by motor deficiencies (slowness of movement, tremors, and postural instability) (Kalia and Lang, 2015), which are caused by degeneration of dopaminergic (DA) neurons (dopamine-producing neurons) in a specific area of the brain called the substantia nigra pars compacta (SNpc). However, the mechanisms underlying neurodegeneration in PD remain unknown (Kalia and Lang, 2015).

In adult mice, Shh is expressed in the SNpc, as well as in other areas including the ventral tegmental area and the retrorubral field in the ventral midbrain (Gonzalez-Reyes et al., 2012; Alvarez-Buylla and Ihrie, 2014). Shh expression in these regions is necessary for long-term maintenance of DA neurons, and ablation of Shh leads to DA neuron degeneration and causes PD (Gonzalez-Reyes et al., 2012). Activation of Hh signaling protects DA neurons, and treatment with the Smo agonist purmorphamine (Briscoe, 2006; Riobo et al., 2006) attenuates inflammatory responses in cells, thereby blocking DA neuron degeneration (Shao et al., 2017). Detailed mechanistic analyses are critical to clarify these effects.

AD is another common neurodegenerative disorder, with an incidence of 1–3% in the population >65 years of age. AD patients show deterioration of cognitive and memory functions caused by loss of neurons and synapses (Masters et al., 2015). Patients with AD show abnormal elevation and accumulation of amyloid-β, a small neurotoxic peptide cleaved from amyloid precursor protein. Amyloid-β interrupts Shh signaling by distorting primary cilia, suggesting that primary cilia-mediated signal transduction, including the Shh signaling pathway, is attenuated in AD patients (Vorobyeva and Saunders, 2018). In a mouse model of AD and in human AD patients, whereas *Shh* expression is upregulated, Ptch1 and Gli1 signaling is decreased, and impaired neurogenesis occurs (He et al., 2014b). Taken together, these findings suggest that Shh signal plays essential roles for the integrity of the brain.

## Conclusions and Perspectives

Since the identification of *Hh* as a segment polarity gene in a mutant screen in flies by German biologist Christiane Nüsslein-Volhard in 1980 (Nusslein-Volhard and Wieschaus, 1980), more than 8,000 scientific papers related to Hh have been published in a 40 year period according to a simple survey of the PubMed database (https://www.ncbi.nlm.nih.gov/pubmed/). Nevertheless, new functions, mediators, and regulatory mechanisms are continuously being proposed. The Hh signaling pathway continues to attract the attention of researchers not only because of its important roles in biological processes, but also its unique regulatory mechanisms. Hh proteins are cleaved from long polypeptides, and the receptor proteins for Hh are localized in a specific region of the membrane, the cilium. In addition, the bimodality of the transcription factors Gli2/3, which act as repressors in the absence of Hh and activators in the presence of Hh, constitutes an unusual regulatory mechanism. This bimodal regulation involves ubiquitin-mediated cleavage, which further highlights the uniqueness of the transcriptional regulation. In tissues, Hh acts as a morphogen, and target genes are induced in a concentration-dependent manner. Moreover, multiple feedback mechanisms dynamically regulate Hh activity in a temporal manner, which confers variation in cell fates. Distinct target genes are upregulated by the same Hh signal in a context-dependent manner.

The regulatory mechanisms of the Hh pathway are complex, and new mechanisms are continuously being identified. Because each new finding triggers another question, many researchers in various fields including molecular and cell biology, genetics, medicine, biochemistry, protein structure, chemistry, and mathematical biology have chosen Hh as the focus of their research.

Hh proteins are involved in a variety of biological events such as cell differentiation, proliferation, and survival. The fact that multiple processes that are apparently distinct from each other are induced by a single Hh protein should be addressed in the future. Future studies on Hh could focus on the cell type-specific expression levels of each mediator of the Hh signaling pathway. Although there are more than 30 mediators of Hh signaling, the expression levels of these proteins are likely cell type-specific, which may confer variation in the kinetics and responsiveness to the signal. For instance, Hh target genes are expressed within 2 hours in NIH3T3 cells (Humke et al., 2010), whereas expression begins after several hours in neural progenitor cells (Dessaud et al., 2007; Dessaud et al., 2010). Quantitative analyses of the expression levels of Hh signaling mediator genes or proteins may explain the variation in Hh signaling kinetics and its physiological significance.

The mechanisms underlying the cell type specificity of target genes involved in the Hh signaling pathway should be investigated. Although most signaling mediators are common to Shh, Ihh, and Dhh, the downstream genes induced are context-dependent. This variation may be achieved through crosstalk with other signaling molecules, or differences in the transcription factors interacting with Gli or the epigenetic background (chromatin status) of cells. Even in the same neural progenitor cells, early and late progenitor cells show differential responses to the same Shh protein (Sasai et al., 2014). A recent genome-wide loss of function-based screen performed using CRISPR identified positive and negative effectors of Shh signaling (Pusapati et al., 2018b). This screen was performed in NIH3T3 cells, and the potential findings in other cell types are intriguing. Systematic analysis of the expression of Hh target genes in different cells may reveal the mechanisms underlying the diverse roles of Hh signals in different cellular contexts.

Despite extensive research, many mechanisms underlying Hh signaling may remain undiscovered, and cutting-edge approaches, such as chasing single cells or single proteins, computational prediction, and genome-wide functional screens, are warranted to elucidate these mechanisms.

## Author Contributions

NS undertook the groundwork. MT, an expert of cell biology of cilia and ciliopathies, wrote the genetic disease part. TK, an expert of cancer, wrote the cancer part.

## Funding

The work and publication of this article are supported by grants-in-aid from Japan Society for the Promotion of Science (17H03684, 17K19399, NS; 17H05003, MT) and from MEXT (19H04781; NS), and the Joint Research Program of the Institute for Genetic Medicine, Hokkaido University (TK, NS).

## Conflict of Interest

The authors declare that the research was conducted in the absence of any commercial or financial relationships that could be construed as a potential conflict of interest.
